# Clinical Holistic Medicine: A Pilot Study on HIV and Quality of Life and a Suggested Cure for HIV and AIDS

**DOI:** 10.1100/tsw.2004.24

**Published:** 2004-05-11

**Authors:** Søren Ventegodt, Trine Flensborg-Madsen, Niels Jørgen Andersen, Mohammed Morad, Joav Merrick

**Affiliations:** ^1^ The Quality of Life Research Center, Teglgårdstræde 4-8, DK-1452 Copenhagen K, Denmark; ^2^ Department of Public Heath, University of Copenhagen, Copenhagen, Denmark; ^3^ Norwegian School of Management and the Scandinavian Foundation for Holistic Medicine, Sandvika, Norway; ^4^ Division of Community Health, Ben Gurion University, Beer-Sheva, Israel; ^5^ National Institute of Child Health and Human Development, Office of the Medical Director, Division for Mental Retardation, Ministry of Social Affairs, Jerusalem and Zusman Child Development Center, Division of Pediatrics, Ben Gurion University, Beer-Sheva, Israel

**Keywords:** HIV infection, AIDS, quality of life, QOL, philosophy, human development, holistic medicine, public health, Denmark, Israel

## Abstract

This study was undertaken to examine the association between the immunological impact of HIV (measured by CD4 count) and global self-assessed quality of life (QOL) (measured with QOL1) for people suffering from HIV, to see if the connection was large and statistically strong enough to support our hypothesis of a strong QOL-immunological connection through the nonspecific, nonreceptor-mediated immune system, and thus to give a rationale for a holistic cure for HIV. This cross-sectional population study in Uganda included 20 HIV infected persons with no symptoms of AIDS and a CD4 count above 200 mill./liter. The main outcome measures were CD4 count, global QOL measured with the validated questionnaire QOL1, translated to Luganda and translated back to English. We found a large, clinically significant correlation between the number of T-helper cells (CD4) and global self-assessed quality of life (QOL1) (r = 0.57, p = 0.021), when controlled for age, gender, and years of infection. Together with other studies and holistic medicine theory, the results have given rationale for a holistic cure for HIV. We suggest, based on our findings and theoretical considerations, that HIV patients who improve their global QOL, also will improve their CD4 counts. Using the technique of holistic medicine based on the life mission theory and the holistic process theory of healing, we hypothesize that the improvement of QOL can have sufficient biological effect on the CD4, which could avoid or postpone the development of AIDS. A holistic HIV/AIDS cure improving the QOL draws on hidden resources in the person and is thus affordable for everybody. Improving global QOL also means a higher consciousness and a more ethical attitude, making it more difficult for the HIV-infected person to pass on the infection.

## INTRODUCTION

It is well known, but poorly understood, why HIV-infected patients have very different survival rates and some sex workers are immune to the HIV virus[1,2,3]. Very seldom does an HIV-positive person convert to HIV negative and we only know of one case, a patient in France who seemingly was "healed" by Martin Brofmann[4,5]. The Norwegian professor Gunnar Tellnes[6] witnessed a minor positive change seemingly caused by alternative treatment: "I know a patient who was having alternative treatment and his HIV count has gone down since he started on that treatment". CD4 counts, showing the concentration of T-helper cells in the blood, are a good indicator for the stage and seriousness of the HIV virus infection. Several studies have shown associations between different dimensions of quality of life (QOL) and the CD4 counts for HIV-infected persons: health-related quality of life (HRQOL)[7,8], general health disability[9,10], social functioning, pain symptoms[10], functional performance[9] and HIV Overview of Problems Evaluation System (HOPES) have all shown associations with CD4 counts for people with HIV/AIDS. Other studies have proven HRQOL[11,12] and psychological aspects[9] to have no association with CD4 counts. These findings could be explained by the fact that the QOL concept covers many different aspects of life, measured by different questionnaires[13,14]. We use the most simple and general questionnaire, QOL1[15].

The proximity between consciousness and health is not new. It has shown to be beneficial for people to engage in attempts of personal growth, for example, in groups where they can meet and talk about their life and problems. This has shown to postpone the time of death for women with metastasized breast cancer[16] and to postpone the time for the next thromboses for people with coronary restrictions and partial thrombosis[17].

Our organism is equipped with several lines of immunological resistance. Some of these lines apparently are not based on receptor-mediated immune response, and they constitute the nonspecific immune system, highly dependent on the "soft" nonreceptor-mediated, biological information[18,19,20]. The macrophages and similar cells receive their information about what to engulf and present directly from this information source. According to our knowledge of the immune system's evolution, the nonspecific immune system is not the first to appear in multicellular organisms; many data support our hypothesis, essential for the proposed cure, that it is also still the foundation of the highly developed immune system of present-day organisms[18,19].

In our opinion, a well-functioning, nonspecific immune system derives from great inner coherence of the body[21,22,23,24,25,26,27,28,29,30,31,32], which in turn is related to a high order of the intercellular communication locally and globally, which is experienced as a high QOL. Such a harmonic, coherent state can be obtained by consciousness-based, holistic medicine, helping the patient to heal existentially and develop internal and external coherence[20,21,22,23,24,30,31,32,33]. Maybe this is the main reason why some African prostitutes do not contract HIV despite massive exposure to HIV virus or, in other words, due to a well-functioning and extremely effective basic, nonspecific immune system.

Several theories exist on the regulation of the immune system. Biochemical theories point to chemical networks and Jerne-network, while holistic theories center on biological information on the level of the whole organism. It is in this last perspective that we suggest that increasing the QOL, and thus the coherence of the person and the organism, will increase the immunological self-nonself discrimination, and thus the whole immune system's performance.

## METHODS

To test our hypothesis of a connection between QOL and immunity to HIV, one of the authors (TFM) went to Uganda, where a decreasing part of the population (about 7% over 15 years) is infected with HIV[34], in order to collect data. To make it simple, the short, validated, global QOL1 and QOL5[15] questionnaires were chosen. The questionnaires were translated to Luganda and back, with iterative corrections until the translation from Luganda to English was correct, using three different translators. The person responsible for the blood tests at a TASO (the AIDS Support Organisation) center provided the bloodtest data and we collected the QOL data of the patients with their consent for participation in the study, when they were tested.

The survey included 22 HIV-infected persons between 25 and 52 years of age, known to have been infected for 0–8 years; two persons with a CD4 count below 200 mill./liter were excluded from the study as they were suspected to have AIDS, which can compromise the QOL in itself. Only one received ARV (antiretroviral drugs). No one had any visible signs of AIDS or any infectious disease when examined. The persons were randomly chosen as they came by, without any other kind of selection.

## RESULTS

The association between CD4 counts and QOL1 was large and both clinically and statistically significant (see Fig. 1 and Table 1); no significant association was found between CD4 and age ($p$ = –0.055, r = 0.823), but age was correlated to QOL1. The SPSS version of Pearson´s Correlation and linear regression were used to describe the associations and partial correlation was used to control for age, gender, and years of infection. In the survey of these 20 HIV-infected persons, we found a correlation between the number of T-helper cells (CD4) and global self-assessed QOL measured, with QOL1 controlled for age, gender, and years of infection (r = –0.569, $p$ = 0.021, when controlling for the all three), but no significant data were collected with the more complex QOL5 questionnaire (with resemblance to the HOPES questionnaire)[33]. This was due presumably to the small sample (N = 20) as we found the correlation between QOL1 and QOL5 to be r = 0.70, $p$ = 0.001. Even when we controlled for self-evaluated physical health, a factor known always to correlate strongly to QOL, we still observed the tendency (r = 0.37, $p$ = 0.12). Often the CD4/CD8 count was calculated also (N = 14); the correlation between with QOL1 was insignificant here, due presumably to the small numbers.

Quite surprisingly, it seemed that the more globally the QOL was measured, the stronger the statistical connection to the CD4 count. Fig. 1 is generated from using the shortest of all QOL questionnaires, the validated QOL1[15], which measures the self-evaluated QOL with only one question: How would you assess the quality of your life now? Answer: I, very high; II, high; III, neither high nor low; IV, low; V, very low. Using this simple, generic, and global questionnaire, we have found what seems to be one of the strongest psychobiological connections ever seen, between global QOL and CD4.

## DISCUSSION

The life mission theory[36,37,38,39,40,41] states that everybody has a purpose of life or a talent. Happiness comes from living this purpose and succeeding in expressing the core talent in your life. To do this, it is important to develop as a person into what is known as the natural condition, a condition where the person knows him/herself and uses all his/her efforts on achieving what is most important for him/her. The holistic process theory of healing[42,43,44] and the related QOL theories[30,31,32] state that the return to the natural state of being is possible whenever the person gets the resources needed for existential healing. According to the theory, the resources needed are awareness, respect, care, acknowledgment, and acceptance with support and processing in feeling, understanding, and letting go of negative attitudes and beliefs.

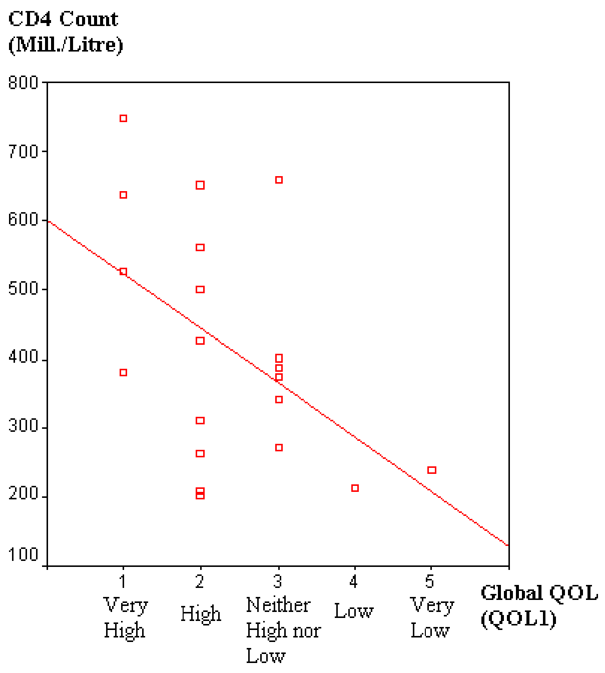

**FIGURE 1**

The precondition for holistic healing to take place is trust, together with the intention of the healing to take place. Existential healing is not a local healing of any tissue, but a healing of the wholeness of the person, making him or her much more resourceful, loving, and knowledgeable of him/herself and his/her own needs and wishes. In letting go of negative attitudes and beliefs, the person returns to a more responsible existential position and an improved QOL. The philosophical change of the person healing is often a change towards preferring difficult problems and challenges instead of avoiding difficulties in life[22,23,24,25,26,27,28,29]. The person who becomes happier and more resourceful often also becomes more healthy, more talented, and able to function[45,46,47].

Holistic therapy is thought to increase coherence and thus to reduce noise and disturbances in the intercellular communications. This, in turn, improves the immunological self-nonself discrimination, which we believe is the main reason for the QOL-CD4 connection. The information system(s) of the body will thus deliver more precise and correct information to the nonspecific immunological cell-lines (the macrophages, Kupfer cells, and others). When QOL is improved, the immune system should — at least in theory — be fine tuned to better eliminate the virus-infected T-helper (CD4) cells, while letting the body's uninfected Th-cells live. This is in accordance with a study showing that QOL actually can predict survival, even if it is not completely clear what causes the high QOL of some patients in this study[48]. Nobody knows yet how effective this holistic cure is against HIV and AIDS, as it still needs to be tested clinically. We have loosely predicted the amount of therapy necessary for healing the HIV infection, from our experience with other patients healing from very serious illnesses in our clinic (see Table 2)[33].

If our assumptions of the meaning of biological coherence for the regulation of the human immune system are correct, the highly sensitive information system of the body might even be able to detect the passive, incorporated DNA fragments incorporated by the HIV retrovirus, making it possible to eliminate the HIV virus completely from the body by apoptosis of the CD4 cells. Presumably, such radical eradication of viruses would require much greater inner coherence than is required to sero-convert and many years of existential therapy.

**TABLE 1**
**Connection Between Global QOL and Number of CD4 Cells (T-Helper Cells) Shown by Partial Correlation and Linear Regression Analysis (SPSS)**

| | Pearson Correlation, Partial Correlation (QOL1) | Pearson Correlation, Partial Correlation (QOL5) |
|---|---|---|
| CD4 | r = –0.486 (*p* = 0.030) | r = –0.114 (*p* = 0.631) |
| CD8 | r = 0.306 (*p* = 0.287) | r = 0.380 (*p* = 0.180) |
| CD4/CD8 | r = –0.344 (*p* = 0.228) | r = –0.178 (*p* = 0.542) |
| CD4 controlled for age and gender | r = –0.565 (*p* = 0.018) | r = –0.300 (*p* = 0.242) |
| CD4 controlled for number of years infection (known years) | r = –0.505 (*p* = 0.028) | r = –0.137 (*p* = 0.577) |
| CD4 controlled for self-assessed physical health | r = –0.373 (p = 0.118) | r = 0.376 (*p* = 0.113) |
| CD4 controlled for age, gender, and number of known years of infection | **r = –0.569 (*p* = 0.021)** | **r = –0.367 (*p* = 0.163)** |

**Coefficients[a]**

| Model | | Unstandardized Coefficients | | Standardized Coefficients | | |
|---|---|---|---|---|---|---|
| | | B | Std. Error | Beta | t | Sig. |
| 1 | (Constant) | 680,379 | 254,065 | | 2,678 | ,018 |
| | sex | -115,595 | 83,534 | -,338 | -1,384 | ,188 |
| | age | 2,953 | 5,803 | ,135 | ,509 | ,619 |
| | Number of years infected | 19,022 | 19,590 | ,214 | ,971 | ,348 |
| | Q6 | -105,977 | 40,901 | -,622 | -2,591 | ,021 |

a. Dependent Variable: CD4

A study of only 20 persons from Uganda, which is very different from our western setting, might be too small a study for reliable hypothesis, but together with similar results from other studies, we believe that the QOL-CD4 connection is to be trusted. Finding a large, significant connection in such a small group means that we have a tendency of large clinical significance. The participants were representative of Uganda's population, as such a large part of the population is infected. Another very important fact was that we looked at a group of people not receiving ARV, which is disturbing the picture in many other studies. The disturbance of the medicine is both on the biological and measurement level because of the use of health-related questionnaires often including the symptoms expected to be alleviated, instead of the more neutral generic and global questionnaires used in our study.

The presented data suggested that global QOL has a strong connection with the progression of the HIV virus infection. Since this connection also has been proven in several other studies, there is fair reason to believe that people who radically improve their QOL, as seems to be possible over a short period of time[43,44,45,46,47,48], will enhance their immunological status and improve their CD4 count.

**TABLE 2**
**A Holistic Cure for HIV: Improving Global QOL Might Improve the CD4 (T-Helper Cell) Blood Count**

| Stage | HIV Status | Holistic Therapy | Immunological Status and Degree of Personal Development |
|---|---|---|---|
| 1 | HIV↑+/AIDS | None | Many inner conflicts that prevent inner coherence and disturb the nonspecific line of the immune system. |
| 2 | HIV+ | 6–12 months | Few inner conflicts, signifying HIV+ without reduced immunity (AIDS). This individual has processed the first 100 gestalts during intensive holistic therapy. |
| 3 | HIV↓+? | 12–24 months | Few antibodies, which shows that the nonspecific immune system catches most cells expressing the HIV virus. This individual is aware of his purpose of life and happy about his life and personal character. |
| 4 | HIV–, but still infected | 24–36 months | Great inner cohesion allows the nonspecific immune system to catch most cells before they express the HIV virus. This individual knows his purpose of life and lives accordingly a life with high quality. |
| 5 | HIV–, not infected, resistant to recontagion | 6 years? | Apoptosis of cells with DNA with HIV virus incorporated as a function of a perfectly fine-tuned biological information system. This individual consistently acts out his purpose of life and is living his social dreams — he has accomplished his social utopia. |

An important argument against the proposed cure is that sufficient improvement in QOL, to be of major importance for the HIV patients, is seldom seen in QOL intervention studies. This makes the possibility for complete sero-conversion to HIV negative unlikely unless the patient is highly motivated for personal change. Even if a sero-conversion made the patients unable to pass the HIV virus on, this situation might not be stable. Such an HIV-negative patient is not cured of the HIV infection, as the retrovirus remains incorporated in the DNA of the T-helper cells throughout life.

The most important aspect of a psychosomatic HIV/AIDS cure is that it is affordable for the poor people of the world, who cannot afford the antiviral drugs. Another very important aspect is that improving global QOL always means a higher consciousness and a more ethical attitude[23,29,41], making it a bigger and more visible problem for the HIV-infected person to pass the infection on. The development of the consciousness of the infected people might be what it takes to puts an end to our global HIV epidemics, the most serous public health problems in the world today.

## CONCLUSION

In a small survey of 20 HIV-infected persons from Uganda, we found a statistically significant connection between the number of T-helper cells (CD4) and global self-assessed QOL.

We suggest, based on this finding and theoretical considerations, that HIV patients who improve their global QOL, can improve their CD4 values and thus avoid or postpone the development of AIDS. The rationale for the cure is found in holistic medical theory, the recently published life mission theory, and the holistic process theory of healing, according to which general health can be improved when the patients improve their QOL. The proposed cure is essentially about helping the patients to improve their QOL and thus their internal coherence. This is on the cellular level equivalent to improving the intercellular communication, which supports the immune system and thus improves the patient’s immunological status. We hypothesize that this can be done to such an extent that the patients gradually improve their CD4 count.

As a result of our experience with holistic therapy based on the life mission theory and the holistic process theory of healing, we have defined a scheme for the connection between HIV status and the improvement of QOL by personal development raising the internal coherence. A holistic HIV/AIDS cure improving the QOL draws on the hidden resources of the person and thus is affordable for everybody. Improving global QOL also means a higher consciousness and a more ethical attitude, making it more difficult for the HIV-infected person to pass the infection on.

The development of the consciousness of the infected people might be what it takes to puts an end to our global HIV epidemic.

## ACKNOWLEDGMENTS

We thank TASO, Entebbe, for their kind assistance. This study was supported by grants from IMK Almene Fond. The quality of life research was approved by the Copenhagen Scientific Ethical approval: Copenhagen Ethical Committee under number (KF)V.100.2123/91.

## BIOSKETCHES

**Søren Ventegodt, MD,** is the Director of the Quality of Life Research Center in Copenhagen, Denmark. He is also responsible for a Research Clinic for Holistic Medicine in Copenhagen and is a popular speaker throughout Scandinavia. He has published numerous scientific or popular articles and a number of books on holistic medicine, quality of life, and quality of working life. His most important scientific contributions are the comprehensive SEQOL questionnaire, the very short QoL5 questionnaire, the integrated QOL theory, the holistic process theory, the life mission theory, and the Danish Quality of Life Research Survey, 1991–94 in cooperation with the University Hospital of Copenhagen and the late pediatric professor Bengt Zachau-Christiansen. E-mail: ventegodt@livskvalitet.org. Website: http://www.livskvalitet.org.

**Trine Flensborg-Madsen**, **MD**, is a research assistant at the Quality of Life Research Centre in Copenhagen, Denmark. At this time, she is finishing her Masters Degree in Public Health at the Department of Public Health, University of Copenhagen. E-mail: tfm@livskvalitet.org

**Niels Jørgen Andersen, MSc,** Professor, Department of Innovation and Economic Organization, Norwegian School of Management. This department conducts research and provides teaching in central topics related to innovation, business development, management of global companies, business history, and economic organization. Research activities within the Department are related to four core subjects within the discipline: business history, cooperative organizations, business development and entrepreneurship, and finally studies of industries with a special focus on the electricity industry. He is also the dynamic chairman of the nonprofit organization Stiftelsen Holistisk Medisin Scandinavia that aims to support the scientific development, research, and documentation of complementary and holistic medicine in Scandinavia. E-mail: niels.j.andersen@bi.no. Website: www.bi.no/users/fgl93013/

**Mohammed Morad, MD**, is Specialist in Family Medicine, Lecturer in Family Medicine at the National Institute of Child Health and Human Development, Division of Community Health, Ben Gurion University of the Negev and the Medical Director of a large area clinic in the city of Beer-Sheva. He has publications on Bedouin health, health aspects, spiritual health, and aging in persons with intellectual disability, and is a presenter on topics such as health policy and services for the disadvantaged at national and international conferences. E-mail: morad62@barak-online.net

**Joav Merrick, MD, DMSc,** is Professor of Child Health and Human Development affiliated with the Zusman Child Development Center, Division of Pediatrics and Community Health at the Ben Gurion University, Beer-Sheva, Israel; the Medical Director of the Division for Mental Retardation, Ministry of Social Affairs, Jerusalem; and the Founder and Director of the National Institute of Child Health and Human Development. He has numerous publications in the field of child and human development, rehabilitation, intellectual disability, disability, health, welfare, abuse, advocacy, quality of life, and prevention. Dr. Merrick received the Peter Sabroe Child Award for outstanding work on behalf of Danish Children in 1985 and the International LEGO-Prize ("The Children's Nobel Prize") for an extraordinary contribution towards improvement in child welfare and well being in 1987. E-mail: jmerrick@internet-zahav.net. Website: www.nichd-israel.com
